# Erratum

**DOI:** 10.1002/jcla.23954

**Published:** 2021-08-18

In Zhang et al.,^1^ the following information was omitted.

Yun Zhang and Qianfeng Yan are joint first authors for this article.

The publisher apologises for omitting this information.
